# Systemic Inflammation Associated with Immune Reconstitution Inflammatory Syndrome in Persons Living with HIV

**DOI:** 10.3390/life11010065

**Published:** 2021-01-18

**Authors:** Caian L. Vinhaes, Mariana Araujo-Pereira, Rafael Tibúrcio, Juan M. Cubillos-Angulo, Fernanda O. Demitto, Kevan M. Akrami, Bruno B. Andrade

**Affiliations:** 1 Instituto Gonçalo Moniz, Fundação Oswaldo Cruz, Salvador 40296-710, Brazil; caianleal@gmail.com (C.L.V.); araujopereira.mariana@gmail.com (M.A.-P.); rafael.santos@aluno.bahia.fiocruz.br (R.T.); j.cubillosangulo@gmail.com (J.M.C.-A.); Kevan.akrami@gmail.com (K.M.A.); 2 Multinational Organization Network Sponsoring Translational and Epidemiological Research (MONSTER) Initiative, Salvador 40210-320, Brazil; fernandademitto@gmail.com; 3 Bahiana School of Medicine and Public Health, Bahia Foundation for the Development of Sciences, Salvador 40290-000, Brazil; 4 Faculdade de Medicina, Universidade Federal da Bahia, Salvador 40110-100, Brazil; 5 Divisions of Infectious Diseases and Pulmonary, Critical Care and Sleep Medicine, Department of Medicine, University of California, San Diego, CA 92093, USA; 6 Curso de Medicina, Centro Universitário Faculdade de Tecnologia e Ciências (UniFTC), Salvador 41741-590, Brazil

**Keywords:** systemic inflammation, mycobacteria, HIV, immune reconstitution inflammatory syndrome (IRIS)

## Abstract

Antiretroviral therapy (ART) has represented a major advancement in the care of people living with HIV (PLWHH), resulting in significant reductions in morbidity and mortality through immune reconstitution and attenuation of homeostatic disruption. Importantly, restoration of immune function in PLWH with opportunistic infections occasionally leads to an intense and uncontrolled cytokine storm following ART initiation known as immune reconstitution inflammatory syndrome (IRIS). IRIS occurrence is associated with the severe and rapid clinical deterioration that results in significant morbidity and mortality. Here, we detail the determinants underlying IRIS development in PLWH, compiling the available knowledge in the field to highlight details of the inflammatory responses in IRIS associated with the most commonly reported opportunistic pathogens. This review also highlights gaps in the understanding of IRIS pathogenesis and summarizes therapeutic strategies that have been used for IRIS.

## 1. Introduction

Globally, nearly 38 million people are living with HIV (PLWH) [1]. The most critical advancement in this epidemic was the development and increased access to antiretroviral therapy (ART), which led to significant reductions in morbimortality through immune reconstitution and attenuation of homeostatic disruption [2]. This has reduced the incidence and severity of opportunistic infections (OI) such as *Mycobacterium tuberculosis* (Mtb) and *Avium complex* (MAC), *Cytomegalovirus* (CMV), Kaposi sarcoma-associated herpesvirus (KSHV), hepatitis C (HCV) and B (HBV) virus, *Cryptococcus neoformans*, *Pneumocystis jirovecci* and *Toxoplasma gondii*. However, paradoxically in a subset of PLWH, ART initiation may trigger clinical worsening with pathologic immune activation against these OIs, characterized by uncontrolled cytokine production known as immune reconstitution inflammatory syndrome (IRIS) [2].

IRIS is defined as a condition occurring shortly after ART initiation (up to 3 months) marked by rapid clinical deterioration with uncontrolled inflammatory processes despite suppression of HIV viral load and increases in CD4^+^ T-cells [3]. It may arise in the setting of a wide variety of pathogens and diverse pathological processes, occurring in two scenarios: (i) unmasking a clinically silent OI (unmasking IRIS); or (ii) paradoxically, deteriorating clinical status after ART introduction despite preceding pathogen-specific therapy and an initial clinical improvement (paradoxical IRIS).

The pathophysiology and immunopathology of IRIS remain unclear, though it is believed to involve an interplay between regulation of restored immune system cells, type and burden of inciting pathogen, changes in T-helper (Th) cells profile and host genetic susceptibility [3]. HIV-associated systemic and persistent background inflammation may independently lead to inadequate regulation of inflammatory activation, thereby contributing to systemic homeostatic disruptions [4,5].

In this review, we will detail IRIS development in PLWH via a thorough examination of the inflammatory process from the progression of HIV infection to respond to infections with the most common opportunistic pathogens associated with IRIS. In this way, we will identify gaps in the understanding of IRIS to highlight possible pathogen-specific targets that may offer the future study of therapeutic interventions in PLWH at risk for IRIS.

## 2. Inflammatory Activation in Persons Living with HIV and IRIS Development

Chronic systemic inflammation (SI) is characterized by persistent activation of both immune and non-immune cells, mainly driven by underlying infectious or inflammatory processes [4]. Chronic SI disrupts the well-coordinated mobilization of immune responses leading to unregulated immune activation and homeostatic disruption. Notably, chronic SI is associated with viral persistence in PLWH and may lead to a persistent inflammatory background and the development of noninfectious comorbidities, such as age-related noninfectious comorbidities (NICMs) and IRIS [6].

**Figure 1:**
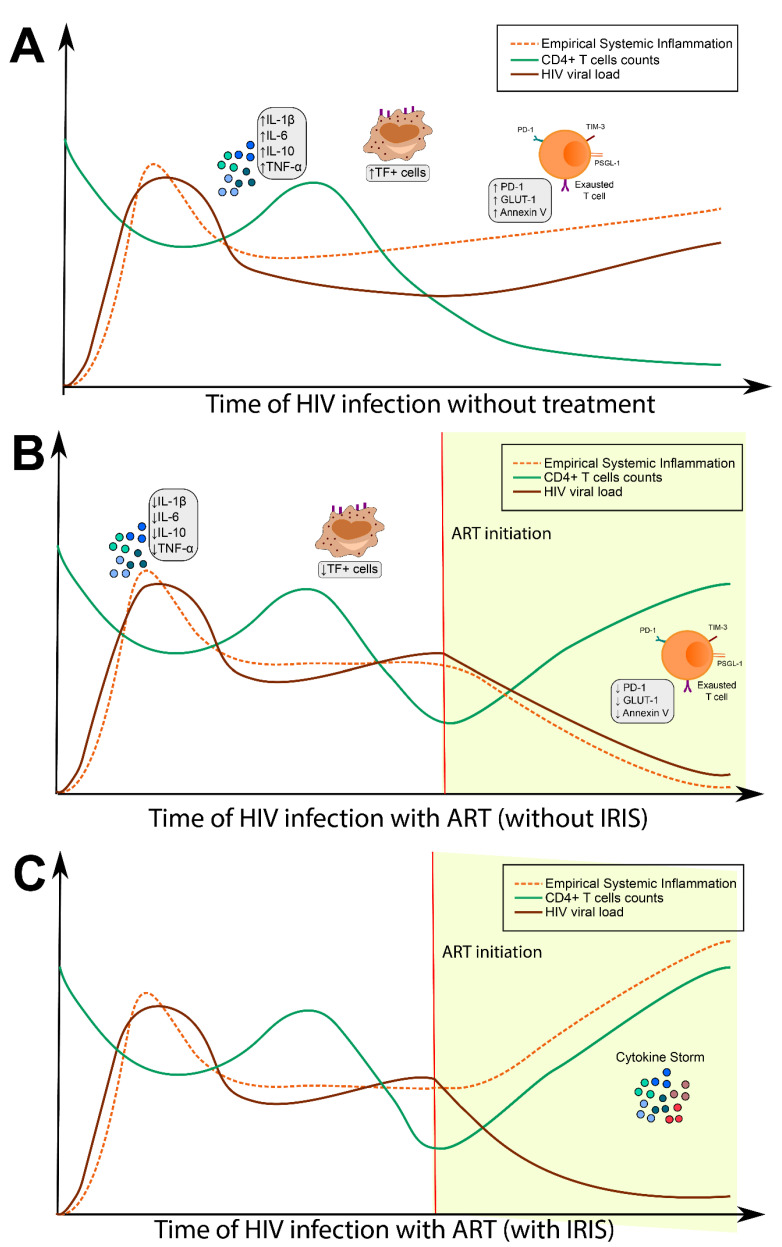
Influence of antiretroviral therapy (ART) on systemic inflammation. (**A**) After HIV infection, patients exhibit high viral loads with a concomitant decrease in CD4^+^ T cell counts. Over the course of HIV infection, people living with HIV (PLWH) experience dysfunction in immune activation and T cell exhaustion, resulting in chronic systemic inflammation and coagulopathy. (**B**) Upon ART commencement, a gradual restoration of the antiviral immune responses occurs, resulting in increased CD4+ T cell counts and decreases in HIV viral load. This process is followed by diminished systemic inflammation and therefore improved prognosis of PLWH. (**C**) With immune reconstitution inflammatory syndrome (IRIS), there is initial clinical improvement, followed by significant deterioration marked by an increased level of inflammation.

Prior work indicates that, in the absence of treatment, HIV-driven SI is associated with increased systemic levels of proinflammatory cytokines such as interleukin (IL)-6, tissue necrosis factor (TNF)-α, and IL-1β [7,8]. After ART initiation, most individuals have a pronounced decline in some circulating cytokine concentrations, while other markers such as IL-6 and C-reactive protein (CRP) remain elevated [8,9]. While reconstitution of the immune system through ART is critical to the reduction of mortality in PLWH, uncontrolled inflammation through the development of IRIS may rapidly lead to clinical deterioration (Figure 1).

### 2.1. Innate and Adaptive Immune Activation: Relationship with Progression of HIV

In an attempt to eradicate HIV infection, the host activates immune responses, particularly innate and adaptive cells, which play a critical role in HIV immunopathology [10,11]. Emerging evidence suggests that several factors contribute to the activation of immune cells, including persistent HIV replication, co-infections and microbial translocations [12].

Several components of HIV-1 trigger innate immune cell activation [13]. The toll-like receptors (TLR) play a pivotal role in HIV-mediated cell activation of monocytes and myeloid dendritic cells responses to TLR8 stimulation, correlating with surrogate markers of disease progression, such as HIV plasma load and CD4^+^ T-cells counts [14]. Conversely, responses of TLR7-stimulated plasmacytoid dendritic cells do not correlate with markers of chronic infection [14]. Using a systems biology approach, Brown et al. identified a TLR independent way to convert macrophages to a proinflammatory state in HIV-1 infection [15].

The consequence of adaptive immune cell activation in HIV progression has been extensively explored. A pioneering study showed high CD38 expression on CD8^+^ T cells as a biomarker for progression to acquired immunodeficiency syndrome (AIDS) and increased risk of death [16]. Another longitudinal study in PLWH before and after ART found a distinct pattern of T-cell turnover in CD4^+^ and CD8^+^ T cells in response to subjacent immune activation [17]. Furthermore, a recent publication has suggested an association between human leukocyte antigen (HLA)-DR and CD38 expression in CD4^+^ T cells with chemokine receptor (CXCR)4-tropism, providing insights into how cellular activation supports R4-switch in later stages of HIV progression [18]. It has been well established that ART counteracts the effects of exaggerated CD4^+^ and CD8^+^ activation, resulting in levels comparable to those individuals without HIV [19]. Despite this knowledge, the immunological nuances of differential activation between CD4^+^ and CD8^+^ T cells have only recently begun to be elucidated [20].

Increased proinflammatory biomarkers resulting from HIV-driven activation of the immune system is well established [12]. A longitudinal multicenter study of 398 PLWH from five African countries revealed that ART-naïve individuals exhibit high levels of inflammatory markers, including C-X-C motif chemokine ligand (CXCL)10, sCD163, sCD14, lipopolysaccharide-binding protein (LBP), IL-6, C–C motif chemokine ligand (CCL)2, and CRP. This pattern remained even after ART initiation, suggesting persistent activation of innate immune cells [21].

### 2.2. Exhaustion of T Cells and Contribution to Immune Dysregulation

Persistent antigen exposure results in cellular exhaustion of effector T cells, characterized by reduced proliferation and cytokine secretion capacity concurrent with upregulation of inhibitory receptors (e.g., programmed cell death receptor (PD)1, cytotoxic T-lymphocyte-associated protein (CTLA)-4, lymphocyte-activation gene (LAG)-3 and CD160 [22,23]. Several studies support the notion that T cell exhaustion is an evolutionary adaptation to protect against chronic SI-related tissue damage and the development of autoimmune diseases [24]. Nevertheless, in the context of HIV infection, both viral antigen exposure and HIV-related immune activation drives T cell hypo-functionality. This, in turn, leads to virus-specific T cell exhaustion that hinders control of HIV replication and contributes to the maintenance of latency reservoirs [25].

Day et al. reported that HIV-infected individuals displayed increased expression of PD-1 and identified an association between the frequency of PD-1^+^ CD4^+^ T-cells and disease progression [26]. Moreover, another study showed that ART initiation does not change expression levels of PD-1 on CD8^+^ T cells, resulting in infective T cell-specific responses against HIV infection [27]. Hoffmann et al. showed that the frequency of CD8^+^ T-cells expressing PD-1/CD38 was linked to an elevated number of viral copies in plasma and lower CD4^+^ T-cell count [28]. Furthermore, PD-1^+^TIM3^+^ CD8^+^ T cells were associated with reduced CD4^+^ T-cell counts in ART-naïve individuals, suggesting a critical role of exhaustion-associated factors in CD4^+^ T cell turnover [29]. Taken together, these findings support the notion that T cell exhaustion plays a pivotal role in the course of HIV infection, contributing to ineffective immune responses, clinical progression and may impact reconstitution after ART initiation.

### 2.3. Metabolic Consequences of Chronic Inflammation Associated with HIV Infection

With homeostatic disruption, during infection and inflammation, immune cells must redirect their metabolic pathways to meet different catabolic and anabolic demands [30,31,32]. Such repurposing of metabolic requirements is crucial to maintain homeostasis and proper immune cell function [33]. HIV-1 infection causes alteration of target cell immunometabolism, thereby reducing adequate metabolic fitness and leading to increased susceptibility to other infections [34].

Numerous studies found that HIV-infected CD4^+^ T cells undergo a metabolic switch from the highly adenosine triphosphate (ATP) yielding oxidative phosphorylation (OXPHOS) pathway to the less energy efficient process of aerobic glycolysis [35,36,37]. Palmer et al. demonstrated that CD4^+^ T cells from HIV-infected individuals possess a higher surface expression of glucose transporter 1 (GLUT-1) that is sustained despite ART [38]. Of note, chronic SI, along with increasing glucose demands, promotes CD4^+^ T cell depletion via a process of metabolic exhaustion [39]. Furthermore, the levels of GLUT-1 expression were correlated with both CD4^+^ T cells activation and depletion.

It is clear that there is a critical association between HIV-driven chronic SI, alterations of cellular immunometabolism, and development of non-AIDS-defining diseases [40], such as HIV-associated neurocognitive disorder (HAND), chronic kidney diseases (CKD) and cardiovascular diseases (CVD). HIV infection is associated with the development of metabolic syndrome that increases the risk of developing CVD and Type 2 diabetes [24]. Notably, adipose-tissue derived cytokines (Adipokines) may contribute to the establishment of chronic HIV/ART-related inflammation and metabolic syndrome, as evident by the correlation of plasma triglycerides with markers of disease progression such as CXCL10 and sIL-2R in untreated individuals. Furthermore, ART alone may only minimally restore the immunometabolic pathways after 12 months of treatment [41], suggesting that persistent changes in the immunometabolism in HIV infection may require alternative therapeutic targets beyond ART.

### 2.4. Hypercoagulation as a Consequence of Persistent Immune Activation

Growing evidence indicates that PLWH is at increased risk of noninfectious chronic complications than their non-HIV counterparts [6], with CVD representing the primary comorbidity among PLWH despite viral suppression and immune reconstitution with ART [42]. It appears that HIV-driven persistent inflammation may result in excessive blood clotting (i.e., hypercoagulation) [43] through activation of both immune and non-immune cells in the course of the infection. Pathologically, HIV-related hypercoagulation is associated with diminished capacity of natural anticoagulants, persistently elevated levels of circulating phospholipids and elevated D-dimer levels, suggesting thrombosis/lysis at an accelerated rate. Additionally, HIV infection appears to lead to an increased platelet activation state, reflected by the augmented activity of the von Willebrand factor, contributing to the hypercoagulation in PLWH [44]. HIV infection is characterized by a chronic inflammatory activation, and one of the consequences of this process is vascular damage, which contributes to the development of hypercoagulation via enhanced tissue factor (TF) activity and reduction in the anticoagulant responses [45]. TF is a cell surface glycoprotein responsible for the initiation of the extrinsic coagulating cascade culminating in the accumulation of factor Xa, fibrin, and thrombin [46]. We have previously identified a subset of CD14^+^ monocytes exhibiting elevated expression of TF in the peripheral blood of HIV-infected individuals, suggesting that a subpopulation of TF^+^ monocytes are critical to the initiation of the coagulate cascade in a factor X/protease activated receptor-1 (PAR-1) dependent manner and contributed to SI via the production of proinflammatory cytokines [47].

## 3. Opportunistic Infection in HIV and Its Impact on the Inflammatory Activation

**Figure 2:**
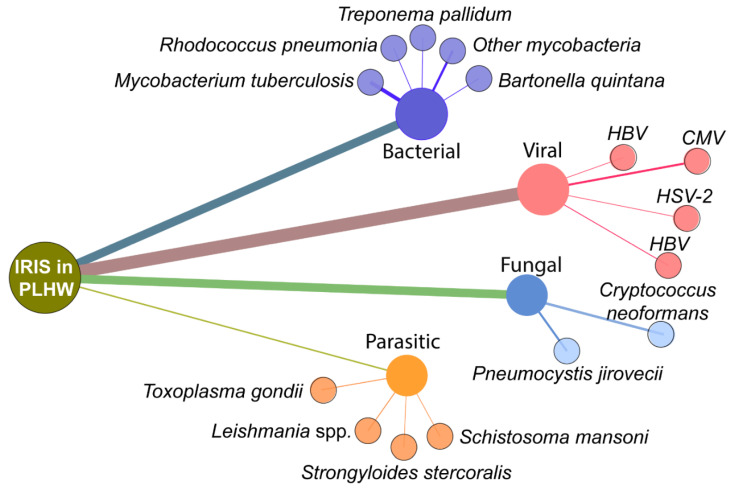
Most common opportunistic infections related to IRIS in PLWH. The figure shows a network analysis of the published studies on IRIS in PLWH available through the NCBI Pubmed Database. The terms used were “immune reconstitution inflammatory syndrome” AND “HIV”. Nodes represent the type of pathogen and genus/species, while the size of edges represents the number of publications retrieved in the search (Date of the search: 10 November 2020).

The natural history of HIV infection leads to depletion of CD4^+^ T-lymphocytes with a reduction in immune capacity [5], allowing for reactivation of inapparent latent infections by dormant pathogens and increased susceptibility to new exogenous infections (reviewed in [48]). The inflammatory milieu of dysregulated immune responses of PLWH to an opportunistic pathogen combined with immune reconstitution after ART initiation creates the perfect environment for IRIS development, though it may be circumvented as early therapy appears to lead to better prognosis and decrease IRIS incidence [49,50,51]. OI often represents the first clinical manifestation of HIV infection depends on the degree of immunosuppression and includes bacterial, viral, fungal and protozoal infections. Infection with *Mycobacterium* is the most common OI associated with IRIS occurrence based on published studies (Figure 2).

### 3.1. Mycobacterial Co-Infection

Among co-infections in PLWH, mycobacteria are the most common OI, primarily by Mtb. In 2019, the World Health Organization (WHO) estimated 456,426 new cases of tuberculosis (TB) among PLWH with 208,000 deaths [52]. The second *Mycobacterium* of interest in PLWH is MAC. Given challenges in diagnosis, the actual incidence of MAC in PLWH varies widely in the literature. A previous study found an incidence of MAC co-infection ranging from 6% to 43% in PLWH [53,54]. Clinical manifestations of co-infection Mtb co-infection vary widely, while previous studies describe lymphadenopathy as the main manifestation in MAC-IRIS. However, we recently found pulmonary manifestations in a cohort of 15 MAC-IRIS participants [55]. Curiously, if mycobacteria disseminate with multifocal lesions, PLWH may manifest multifocal IRIS with immune restoration following ART implementation [56]. This highlights the following pillars of HIV-Mtb co-infection and risk factors of IRIS occurrence: (i) degree of immunosuppression will determine the intensity of immune restoration after ART initiation [57,58,59,60], (ii) mycobacterial dissemination with extrapulmonary lesions [58], (iii) dynamic with other opportunistic infections [48,58].

**Figure 3:**
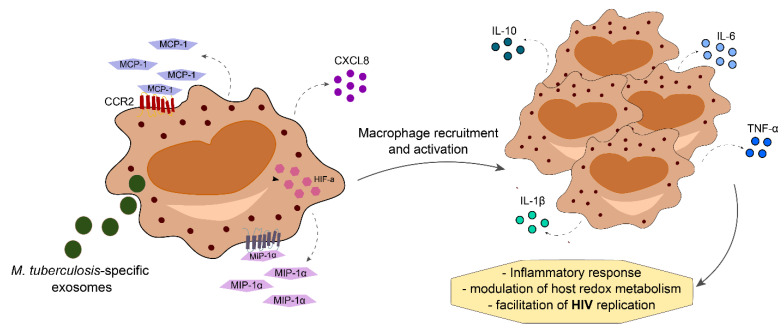
*Mycobacterium tuberculosis*-HIV co-infection increases levels of cytokine and chemokines produced by activated macrophages, leading to macrophage recruitment to the site of infection. This mechanism increases the production of proinflammatory mediators, thereby increasing inflammatory responses with modulation of host redox metabolism that ultimately facilitates HIV replication.

Co-infection with pathogenic mycobacterium can lead to increased SI in PLWH. TB infection itself leads to changes in inflammatory proteins and lipid mediators that persist even after completion of antitubercular therapy, culminating in intense inflammatory imbalance [61,62]. Mtb-specific exosomes increase the expression of CXCL8, CCL2, macrophage inflammatory protein (MIP)-1ɑ and hypoxia-inducible factor (HIF)-1 in monocytes of PLWH. Consequently, this process promotes HIV replication through modulation of host redox metabolism, promoting lymphocyte and macrophage-mediated inflammatory response [63]. In comparison to individuals with mono-infections, those with HIV-TB co-infection express higher levels of inflammatory cytokines such as IL-6, TNF-ɑ, IL-10, and IL-1-β (Figure 3) [64,65,66,67]. As a consequence of chronic SI, anemia may develop and impact treatment success with antitubercular and antiretroviral therapy, in addition to the risk of IRIS development (reviewed in [68]).

The immunopathogenesis of IRIS in patients with tuberculosis (TB-IRIS) has been extensively studied and seems to depend on myeloid activation. In an immunocompetent host, myeloid cells have two signals to become fully activated: (i) recognition of microbial products by pattern recognition receptors and (ii) interaction with interferon (IFN)-γ produced by CD4^+^ T-cells (reviewed in [50]). Importantly, IFN-γ production triggers full macrophage activation with the production of a proinflammatory response to contain mycobacterium infection and prevent dissemination [50,69]. By contrast, an immunosuppressed host may have temporal uncoupling of the innate and adaptative immune response, with only the first signal for myeloid activation, thereby lacking full activation of this lineage. With incomplete activation of macrophages, unchecked *Mycobacterium* proliferation occurs, leading to a significant increase in mycobacterial burden with the risk of disseminated disease [70]. Intriguingly, the absence of macrophage activation leads to an increased number of primed macrophages accumulated in host tissues, creating a state of immunological hyperresponsiveness to CD4^+^ T-cells [50,71]. With persistent immune suppression, mycobacterium tends to proliferate with dissemination to other tissues, most commonly lymph node and pleura, with a consequent increase in macrophages without full activation. After ART initiation, rapid hyperactivation of T-cells by mycobacterium specific antigens [72,73,74,75] leads to activation of mycobacterium-primed macrophages, accumulated in tissues, with second myeloid activation driving a sudden spike in inflammation and subsequent cytokine storm leading to the tissue damage [50,76] that characterizes mycobacterial IRIS (M-IRIS).

On the molecular level, full myeloid activation resulting from CD4^+^ T-cell IFN signal leads to a cytokine storm orchestrated by monocyte and macrophage populations with a higher production of matrix metalloproteinases (MMPs) [77,78]. The imbalance between MMP levels and tissue inhibitors of metalloproteinase (TIMPs) outpaces collagen deposition leading to tissue remodeling [78]. Importantly, MMP/TIMP dysregulated production was associated with cavitation and, consequently, bacterial proliferation [79], which could cause suppurative manifestations.

Mycobacterial IRIS (M-IRIS) can occur in two forms: unmasking, when there is a flare-up of an underlying, previously undiagnosed infection soon after ART is started; or paradoxical, where there is worsening of a previously treated infection after ART is started [3,80]. Unmasking and paradoxical IRIS may exhibit different profiles of inflammation. Comparing both populations, Haddow et al. [81] showed that paradoxical patients present innate immune response activity, with lower values of monocyte and regulatory T-cell activity markers, whereas unmasking patients present higher levels of these markers and a greater level of IFN-γ than expected against mycobacterium.

While studies evaluating the incidence of unmasking TB-IRIS are scarce, it may be possible to predict which group of patients are at the greatest risk. According to Lawn et al. [82] the chance of a PLWH with CD4^+^ T lymphocyte levels <200 cells per μL to develop unmasked TB is about 40% (95% CI 6–61%). The frequency of TB infection in the asymptomatic HIV-infected population and the degree of immunosuppression before ART is closely linked to the risk of unmasked TB-IRIS [83].

About two-thirds of unmasking TB-IRIS forms involve the lung, with severe pulmonary tuberculosis or bronchiolitis obliterans organizing pneumonia [84,85]. In addition, patients generally have a higher viral load and a low CD4^+^ count at the beginning of therapy. The diagnosis of unmasking TB-IRIS, however, is made when these values begin to reverse, with a decrease in viral load and an increase in lymphocyte count [85].

Conversely, paradoxical TB-IRIS can range from 8 to 54% of patients depending on the epidemiological settings [86,87,88]. The development of TB-IRIS is an important factor associated with poor prognosis and mortality in TB/HIV patients. Although the exact trigger for IRIS remains unknown, it is apparent that the frequency of CD14^++^CD16^−^ monocytes is an independent predictor, and is closely linked to levels of CRP, TNF, IL-6 and tissue factor during IRIS [76]. A meta-analysis that analyzed data from 1048 PLWH with TB-IRIS demonstrated that 18% (95% CI: 16–21%) of patients were diagnosed with TB-IRIS before starting antiretroviral treatment and that, among 7% who died, 2% (95% CI: 1–3%) had their deaths associated with IRIS (reviewed in [89]).

The impact of TB-IRIS occurrence on TB treatment outcomes remains unclear. A retrospective study with 292 PLWH showed that almost 21% of TB-IRIS patients presented an unfavorable treatment outcome [88]. However, another study conducted in South India seeking to determine the impact of paradoxical TB-IRIS on TB treatment outcomes found no consistent divergences between those with and without IRIS [88].

Despite improved outcomes in those with mycobacterium-HIV co-infection, Mtb remains a critical challenge in the clinical management of PLWH, requiring ongoing effort to identify gaps in novel therapeutic strategies.

### 3.2. Viral Co-Infections

Several viral pathogens may be associated with IRIS in PLWH with significant immunosuppression. In a recent multicenter cohort study, we found 18 viral IRIS cases among 206 severely immunosuppressed participants recruited in the United States, 9 associated with varicella-zoster virus (VZV), five CMV, two KSHV, one HBV and one HCV [90]. 200 PLWH were recruited from Kenya with 9 cases of viral IRIS, including 6 VZV, 2 KSHV and 1 HBV [90]. Finally, in Thailand, among 100 enrolled participants, 2 CMV IRIS cases were identified [90] importantly, while manifestations of end-organ diseases (EOD) by CMV or oncological manifestations as Kaposi sarcoma, viral IRIS in PLWH remains incompletely understood and may offer distinct pathways in pathogenesis and clinical presentation (reviewed in [48]).

CMV is a herpesvirus that can cause several manifestations in an immunosuppressed host, ranging from localized or disseminated EOD, typically in those with CD4^+^ counts below 50 cells/mm^3^. In an Italian cohort, Lichtner et al. determined that active CMV diseases were associated with twice the increased risk of non-AIDS events and deaths for cardiovascular and cerebrovascular events [91]. HIV-CMV co-infection may lead to T-cell senescence and subsequent negative effects on immune activation, contributing to non-AIDS events [48,92]. Importantly, those with CMV-HIV present with a lower CD4^+^/CD8^+^ ratio than CMV negative PLWH [93], which may contribute to the inadequate immune restoration and increase risk of IRIS following ART. Conversely, CMV replication seems to correlate with the degree of immunosuppression [94], creating a propitious milieu to IRIS development. The definitive diagnosis of CMV-IRIS remains difficult, particularly when the site of manifestation is unusual. Retinitis is the most common manifestation of CMV in PLWH and consequently could occur after ART introduction [95,96], as demonstrated recently by Ruiz-Cruz et al., finding evidence of CMV-immune recovery retinitis (IRR). Among the 55 cases of CMV-IRIS, 35 participants developed unmasking in a median of 4 weeks, whereas 20 paradoxical IRIS occurred in a median of 6 weeks [97]. Curiously, no significant differences before ART introduction were found in total CD4^+^ T-cells count nor CD4^+^/CD8^+^ ratio, and lower CD4^+^ T-Cells count was not associated with increased risk for IRIS [97]. Of note, CMV pneumonia, not typical of primary infection, could occur in the setting of CMV-IRIS, emerging as an important differential diagnosis with the pulmonary presentation of *P. jirovecci* [98].

Another important herpesvirus in the context of HIV infection is human herpesvirus (HHV)-8 or KSHV [99]. In a prospective study with 136 participants, 34.6% of those with HIV-KSHV had sustained inflammatory activation despite HIV virologic suppression [100], with accelerated atherosclerotic diseases associated with increased risk of non-AIDS events [100]. Of note, the process that leads to chronic inflammation may also underly the development of cytokine storm and IRIS after ART (reviewed in [50]).

Hepatitis viral co-infection in PLWH represents another group of viral pathogens associated with significant inflammation. HBV is the leading cause of chronic liver diseases, affecting 7.4% of PLWH worldwide [101]. Importantly, PLWH is at increased risk of chronic HBV infection [102], which leads to an increase in inflammation [103]. In the setting of intense immune activation, ART introduction may trigger a hepatitis flare (HF) as an IRIS manifestation [104,105]. HF in mono-infection is associated with proinflammatory and antiviral immune markers, including TNF-α and IFN-γ [106]. Importantly, higher levels of CXCL10 were found in those with HIV-HBV co-infection who developed HF [105], highlighting the contribution of the IFN pathway in HF-IRIS development. HCV affects 20–30% of PLWH in the United States [107,108], with a shared route of transmission leading to an increased number of co-infections. These patients may manifest complex clinical scenarios with accelerated progression to hepatic fibrosis and higher rates of liver decompensation [109], primarily when CD4^+^ T-cells counts are above 200 cells/mm^3^ [110,111]. Importantly, extrahepatic conditions in HIV-HCV co-infection may occur [112,113], including cardiovascular events [114], possibly resulting from higher activity of tissue factor [115]. Notably, in PLWH and chronic HCV, there is incomplete CD4^+^ T-cell restoration [114] and higher concentrations of proinflammatory markers, including sCD14, IL-6 [116] and IFN-α [117]. This creates an important challenge in the immune reconstitution process, as incomplete recovery leads to poor HCV prognosis even after ART introduction [112]. With the introduction of effective antiviral therapy against HCV, this trend may be reversed in PLWH.

The pattern of immune responses in viral IRIS remains poorly understood (reviewed in [48]) despite the role of viral co-infection in inflammatory activation of PLWH. In a recent study, we demonstrated that patients who develop viral IRIS lack consistent differences in biomarker levels when compared to those without IRIS. However, patients that developed viral IRIS demonstrated a different correlation profile from those that developed mycobacterial IRIS or without IRIS, suggesting that ART initiation in patients that developed IRIS mediated by viral pathogens leads to uncoupling and disorganization of the immune responses characterized by the decreased correlation between biomarkers [90]. A thorough understanding of the pathogenesis and therapeutic management of viral IRIS is critically lacking, hindering both diagnosis and management.

### 3.3. Fungal and Parasitic Co-Infections

Parasitic and fungal co-infections occur in PLWH with severe immune suppression, posing a significant risk of IRIS and include the following: pneumocystosis, cryptococcosis, cryptosporidiosis, histoplasmosis, toxoplasmosis and strongyloidiasis. Sereti et al. found an incidence of 21.7% non-viral and non-mycobacterial IRIS, with 21 cases among 97 total IRIS cases, 8 associated with *Cryptococcus* (C-IRIS) [51].

*Cryptococcus neoformans* is one of the most prevalent OI associated with IRIS, typically occurring in those with CD4 count less than 100 cells/mm. Severe cases of IRIS may occur with the brain and intradural abscesses [118]. Previous studies found that risk for clinical deterioration after ART introduction occurs in those with high cryptococcal antigen loads and lower levels of inflammatory markers (CRP, D-dimer, IL-6 and IL-1RA) found in cerebrospinal fluid (CSF) [119,120]. Jarvis et al. evaluated cytokine responses in HIV-associated cryptococcal meningitis finding that macrophage activation linked to Th1 and Th17 activation in CSF was associated with cryptococci clearance and survival at 2 weeks [121]. Khaw et al. recently demonstrated Th1 mediated brain damage in IRIS by Cryptococcus neoformans in mice [122]. Curiously, this appears associated with an aquaporin critical for brain water flow regulation that depends on Th1 [122]. In another report, Meya et al. showed that aberrant T-cell function leads to subsequent increases in cytokine responses, with a lower frequency of memory subsets of CD4 and CD8 T-cells expressing IL-2, IL-17 and IFN-γ [123]. While others have found that early ART introduction was associated with decreases in non-cryptococcal IRIS incidence, Boulware et al. recently showed that deferring ART for five weeks improves survival in PLWH presenting with cryptococcal meningitis, primarily in those with lower white cell counts in the cerebrospinal fluid [124]. Interestingly, high plasma levels of IL-5 and IL-7, as opposed to CSF levels, may reflect poor clearance of cryptococcus prior to ART initiation. Additionally, IL-7 pathway dysfunction in T-cells was linked to C-IRIS [120,121,125,126]. While elevated inflammatory cytokines in the CSF related to intact Th1, Th2 and Th17 profiles appeared protective against IRIS, elevated MCP-1 and MIP-1α, in addition to higher CD8^+^ T-cells, carried an increased risk of subsequent C-IRIS. Inadequate inflammation may ineffectively clear cryptococcal antigen and result in exuberant inflammation with immune reconstitution and consequentially C-IRIS.

Pneumocystis pneumonia (PJP), caused by the ubiquitous fungus *Pneumocystis jirovecci*, affects 70–80% of PLWH with CD4 T-cell count less than 200 prior to prophylaxis implementation and ART [127]. PJP-IRIS manifests as worsening hypoxia following initiation of ART, with mouse models demonstrating elevated inflammatory cytokine profiles that may be attenuated by IFN-γ signaling of anti-inflammatory Foxp3-positive CD8+ T cells. Adjuvant corticosteroid therapy serves to limit this inflammation in the course of treatment without compromising pathogen clearance by either classically or alternatively activated macrophages prior to full immune reconstitution with ART. Disruption of surfactant with subsequent exposure of proinflammatory moieties may be another pathway for the development of PJP-IRIS in PLWH. PJP-IRIS is associated with severe morbidity, though data remains sparse, with respiratory failure occurring in up to 62.9% of individuals [128].

Another clinically relevant OI is *Toxoplasma gondi*, clinically characterized by mass effect in the central nervous system. The prevalence of anti-*Toxoplasma* antibody among PLWH vary widely around the world, ranging from 11% in the USA up to 80% in European, Latin American and African countries [99,129,130,131] and patients with less than 100 CD4 T-cells/mm^3^ are at higher risk of co-infection or reactivation [132,133,134,135]. IRIS with *Toxoplasma* may present with focal encephalitis marked by headache, fever, confusion and motor weakness [132,133,135] secondary to brain edema triggered by immune reconstitution. Immune mechanisms underlying the development of the extremely rare case of toxoplasma-IRIS remains to be further elucidated in future studies.

Fungal and parasites OI in PLWH could lead to severe clinical deterioration, as reported above. More robust clinical investigations targeting the improvement of the management of PLWH co-infected with these pathogens are warranted.

## 4. Use of Host Markers to Predict IRIS

Host-based markers are extensively being explored to improve predictive and diagnostic tools for IRIS in PLWH who have a concomitant OI [104,136,137,138,139,140]. In this context, several host factors have been studied as an IRIS predictive tool, including genetic markers, immune metabolomic and biomarkers of SI (Table 1).

The genetic factors associated with IRIS occurrence are poorly elucidated, and most of the studies were focused on TB-IRIS. We recently evaluated the role of leukotriene A4 hydroxylase (LTA4H) polymorphism in TB-IRIS [141] (Table 1). Comparing wild genotype individuals with mutant genotypes, we found a higher incidence of severe IRIS among mutant LTA4H genotypes, despite a similar IRIS incidence in both groups [141]. A recent study evaluated the association between HLA-B, HLA-C and killer-cell immunoglobulin-like receptors (KIR) genotypes and TB, HIV-1 infection and TB-IRIS [142]. They identified an increased risk for IRIS among carriers of the KIR2DS2 gene, the HLA-B*41 allele, the KIR2DS1+HLA-C2 pair and the not carriers of the pairs KIR2DL3 + HLA-C1/C2 and KIR2DL1 + HLA-C1/C2 [142] (Table 1). The polymorphisms in cytokine genes that define PLWH who experienced mycobacterial and viral IRIS were studied by Price et al. [143]. They determined a low proportion of IL12B-3′UTR*2 carriers among patients who experienced IRIS associated with herpesvirus compared to higher frequencies in non-IRIS or other IRIS patients [143]. Additionally, it appears that patients with M-IRIS rarely carry IL6-174*C and never carried TNFA-308*2, whereas TNFA-308*2 was present in the majority of those with IRIS-associated herpesvirus [143] (Table 1).

Transcriptomic predictors of C-IRIS were recently demonstrated using blood profiles of 54 PLWH and cryptococcal meningitis from those 27 developed C-IRIS. The results revealed low expression of the interferon pathway and higher expression of transcripts that encode granulocyte responses as predictors of C-IRIS [144]. Importantly, the authors identified a transcriptomic profile associated with early C-IRIS occurrence, characterized by an abnormal upregulation of transcripts associated with innate immunity, whereas those with late C-IRIS expressed an upregulation of transcripts expressed in cells T, B and natural killer [144], highlighting the inclusion of AIM2, BEX1, and C1QB as novel biomarkers for both early and late C-IRIS events [144].

The immuno-metabolomic factors used to predict IRIS are commonly studied with a focus on TB-IRIS. Silva et al., in a pilot metabolomic study of TB-IRIS, enrolled 26 participants with IRIS and 22 HIV-TB coinfected controls. Using a regression model, the authors found that pathways indicating the participation of arachidonic acid, linoleic acid and glycerophospholipid metabolism were relevant to identify individuals at higher risk of TB-IRIS [145].

While studies of host genetic factors are sparse, there is extensive research into which SI biomarkers may aid in the prediction of IRIS. In a cohort by Haddow et al. in South Africa, lower levels of IL-10 and CCL2 were found in individuals that developed paradoxical TB-IRIS, whereas higher CRP levels and IFN-γ were identified in an unmasking presentation [81] (Table 1). A similar result of lower levels of CCL2 was found by Oliver et al. In a study of 15 patients that developed paradoxical and 11 unmasking TB-IRIS cases [146]. Additionally, levels of IL-18 and CXCL10 were higher in paradoxical cases, whereas unmasking participants showed higher levels of IL-18 [146]. The relevance of cytokines in the prediction of paradoxical TB-IRIS was also reaffirmed in a cohort study with participants from Malaysia and India, where patients who developed IRIS had high levels of IL-18 prior to ART [147] (Table 1).

Considering the dynamic process surrounding inflammatory activation, we recently developed a composite score in a multicenter cohort study to predict TB-IRIS [90] (Table 1). First, we identified higher expression of IL-6, IL-10, IL-27, sCD14, IFN-γ, TNF-α, hyaluronic acid and D-dimer with lower hemoglobin levels in IRIS participants. Next, we attributed a score of one point if the biomarker values expression were above the 75th percentile for IL-6, IL-10, IL-27, sCD14, IFN-γ, TNF-α, hyaluronic acid, D-dimer and below of the 25th percentile for hemoglobin levels. We also considered body mass index (BMI) levels and created a composite score ranging from 0 to 10 points comprised of clinical, laboratory and immunological parameters, finding reasonable predictive accuracy for development of M-IRIS in those with scores >3 points (75% by AUC) [90]. Importantly, our composite score demonstrated 100% of specificity in scores above 8, 98.29% above 7 points and 95.1% above 6 points, exhibiting a higher predictive value for mycobacterial IRIS above 6 points [90]. Extending our score to predict viral IRIS, we found an accuracy of 54% for viral IRIS prediction versus no IRIS and 75% in ROC analyses between viral versus mycobacterial IRIS. Of note, the accuracy of our composite score to predict IRIS, regardless of the associated pathogen, was 66% if above 2 points. Another composite score was recently developed by Musselwhite et al. in a prospective study conducted in South Africa and Mexico, using plasma levels of CRP, sCD14, IFN-γ and hemoglobin [148]. ROC analysis reveals the accuracy of 82% to predict TB-IRIS versus no IRIS and 85% in prediction of TB-IRIS versus other-IRIS in those with scores >1.5 points [148] (Table 1).

Extending our efforts to identify MAC-IRIS predictors, we recently evaluated plasma markers and markers associated with lymphocyte activation, including HLA, CD38, Ki67 and PD-1, in a cohort of 15 MAC-IRIS participants. Increased expression of CD38 (frequency or MFI) by CD 8 T-cells were found as risk factors for MAC-IRIS development [55] (Table 1). Additionally, elevated alkaline phosphatase and D-dimer levels support the diagnosis of MAC-IRIS in our cohort [55].

Recently, Mizukawa et al. evaluated inflammatory biomarkers in the prediction of CMV reactivation after immunosuppressive therapy in 45 participants with drug-induced hypersensitivity syndrome or drug reaction with eosinophilia and systemic syndrome and found that higher baseline levels of IL-8, IL-10, IL-12p70, IL-15, G-CSF and CCL2 were associated with the development of CMV reactivation [149] (Table 1). This may aid in the development of prediction tools for CMV-related IRIS using the host inflammatory biomarkers.

Inflammatory markers have also been used to predict the development of C-IRIS. Boulware et al. recently suggested that lower inflammatory activation in CSF, marked by decreased CSF leukocytes, IFN-g, IL-6, IL-8 and TNF-a, in PLWH with cryptococcal meningitis was associated with IRIS [120] (Table 1). Importantly, the cytokine profile found in CSF may distinguish IRIS from the cryptococcal meningitis relapse. Another report conducted by Jarvis et al. identified a protective profile in those PLWH presenting with cryptococcal meningitis, finding that increased macrophage activation in CSF, mainly directed by IL-6, IFN-g, IL-4, IL-10 and IL-27, was associated with survival at two weeks [121]. Higher CSF ratios of CCL2/CXCL10 and CCL3/CXCL10 was also associated with C-IRIS in a prospective study of 128 PLWH [125]. On the other hand, Akilimali et al. argue that plasma but not CSF levels of IL-7 and IL-5 predicts C-IRIS [126] (Table 1).

In a recent retrospective cohort of 287 PLWH coinfected with HBV and/or HVC, 207 with HCV co-infection, 70 with HBV coinfected and 10 with both HBV and HCV co-infection, we evaluated pre-ART markers associated with death and HF [104] (Table 1). We found that the risk of HF is higher in HBV co-infection only and HBV/HCV co-infection than in co-infection by only HCV. Additionally, higher levels of alanine aminotransferase and IL-10 were associated with HF, whereas higher D-dimer, IL-6, IL-8 and sCD14 were associated with death [104].

In summary, considering the immunopathogenesis of HIV and co-infection with various OIs, profiles of host biomarkers of immune activation emerge as an option to identify those at increased risk of IRIS development and may ultimately offer insight into the development of targeted therapies to improve prognosis in PLWH.

## 5. Host-Directed Therapies to Prevent and Treat IRIS in PLWH

Host directed therapies have emerged as a personalized approach to mitigate the unfavorable impact of diseases marked by SI. As IRIS is a condition marked by homeostatic imbalance mediated by a cytokine storm after host immune reconstitution, therapies targeting host responses are growing with a focus on the identification of pharmacological intervention to attenuate SI and prevent tissue damage mediated by IRIS (Table 2).

In severe cases of IRIS, corticosteroids are recommended, though they require adjustment according to the severity. The benefit of corticosteroids may be tempered those at increased risk of steroid-induced complications (hypertension, diabetes mellitus, risk of a new infection, baseline altered mental status) (reviewed in [150]). A randomized placebo-controlled trial demonstrated reduced hospitalization, therapeutic procedures and hastened improvement in symptoms, performance and quality of life in patients with TB-IRIS receiving corticosteroids [151]. A recent sub-study of the pre-ART trial with prophylactic prednisone did not demonstrate long-term benefits in lung function but for improved lung function at week 4, possibly by reducing TB-IRIS [152] (Table 2). Overall, prednisone was well tolerated in these studies and is an effective therapy to reduce or prevent tissue damage in those with paradoxical TB-IRIS.

In contrast, steroid therapy in C-IRIS, particularly meningitis, may increase the risk of incomplete clearance of cryptococcus and recurrent IRIS, with no mortality benefit and increased adverse events [153,154]. This was reinforced in a prospective, double-blind, randomized, placebo-controlled trial [153] that determined that dexamethasone increased rates of disability and was associated with excess severe adverse events, including infectious episodes and renal, gastrointestinal and cardiac disorders.

The International Study of Patients with HIV on Rifampicin ING (INSPIRING) investigated antiviral activity and safety of dolutegravir (DTG) among ART-naive patients with HIV-TB co-infection demonstrating low incidence of IRIS, with 4 (6%) participants in the DTG arm [155] (Table 2). This result is similar to a meta-analysis of PLWH, where IRIS was reported in 1/414 participants on DTG, though no significant difference in the risk of IRIS was found between the study arms [166]. In general, these studies are important and support analysis of drug safety, since DTG is classified as a drug that can produce IRIS (could occur in 0.1–1% of treated patients) as a rare adverse event according to the European Summary of Product Characteristics [167].

Maraviroc is a novel small-molecule CC-chemokine receptor 5 (CCR5) antagonist with potent anti-HIV-1 activity and favorable pharmacological properties [168]. A randomized, double-blind, placebo-controlled clinical trial [156] sought to determine whether maraviroc decreases the incidence of IRIS. Interestingly, while those treated with maraviroc had circulating inflammatory cells expressing CCR5, CCR5 blockade failed to prevent the development of IRIS (Table 2). Similarly, the use of TNF-α inhibitors does not decrease IRIS and may, in fact, increase the risk of IRIS development in a cohort study of pulmonary TB cases from Japan [157] (Table 2). In this study, the authors hypothesize that it is possible that TNF-α inhibitors may be partly associated with IRIS development as they promote a Th2 cytokine-dominant response [157].

Alternative options in the management of paradoxical TB-IRIS are mainly supported by case report evidence. Anakinra, a recombinant IL-1 receptor antagonist [169], may control the inflammatory activation mediated by IL-1, with reduction or discontinuation of steroids in patients at risk of death or serious morbidity [158]. Bevacizumab is a humanized monoclonal antibody against VEGF (reviewed in [170]) used in patients with IRIS who had a marked improvement in both the functional and anatomical outcomes without any deterioration at 12 months follow-up [160]. Additionally, the therapeutic effect of Adalimumab, an anti-TNF monoclonal antibody, was tested in a TB-IRIS case of [161], showing efficacy in IRIS management without compromising control of mycobacterial or HIV infection. Finally, in three patients presenting with steroid-unresponsive mycobacterial IRIS, there was clinical improvement temporally associated with infliximab administration (also an anti-TNF monoclonal antibody), without adverse impact on immune recovery and HIV virologic control [161,171].

Other IRIS events associated with co-infection with viruses (hepatitis B) and fungi (*Cryptococcus*) have a different clinical treatment from TB-IRIS. Thalidomide is a barbiturate-like medication with immunomodulatory, anti-angiogenic and anti-inflammatory effects [172] that offers a promising therapeutic option in those with steroid-refractory IRIS [162,163,173]. In France, treatment with thalidomide in two PLWH with refractory, corticosteroid-dependent and life-threatening paradoxical C-IRIS meningitis resulted in rapid clinical remission [162]. In a retrospective multicenter cohort study in France with 120 PLWH and treated cryptococcosis on ART, two patients received thalidomide for 4 months with a favorable progressive return to normal lymph-node size [163].

Co-infection with HBV responds best to ART that includes an anti-HBV component (tenofovir), leading to decreased viral loads and reduced risk of HF or IRIS [164]. Additionally, in two Japanese patients with HBV-HIV that developed IRIS following the administration of ART, the levels of HIV RNA and HBV DNA decreased [165]. These cases demonstrate that ART with an active component against HBV is critical in all cases of IRIS mediated by HBV co-infection.

The heterogeneity of IRIS in terms of clinical presentation and severity, management requires personalized treatment. Emerging predictive scores to stratify risk of IRIS and identification of possible therapeutic targets are promising though they must be confirmed in future rigorous prospective clinical trials. Until such a time, corticosteroids remain the mainstay of therapy IRIS through immune modulation (thalidomide), and monoclonal antibodies targeting inflammatory components (TNF-alpha inhibition) are emerging as a second option in the cases of steroid-refractory disease.

## 6. Conclusions

HIV infection requires treatment with antiretroviral drugs that can lead to excess systemic inflammation during immune reconstitution. This condition, characterized as IRIS, occurs primarily in cases of co-infection with pathogens including mycobacteria, viruses, protozoa and fungi, with co-infection with Mtb primarily IRIS associated in the era of antiretroviral therapy. Mtb-HIV co-infection remains a critical problem that requires significant efforts and meticulous attention during management. Though IRIS is associated with relatively low mortality, IRIS events may lead to increased morbidity and be compounded by a lack of effective predictive tools and therapeutic strategies. As an unregulated cytokine storm after the onset of ART, IRIS-associated inflammatory biomarkers may aid in predicting the risk of IRIS and target individualized immune-modulatory therapy. Our review aimed to summarize the most recent scientific concepts regarding IRIS using a combined approach of immunological/inflammatory and clinical aspects, thereby increasing the current knowledge of this highly morbid syndrome. IRIS prediction and therapeutic strategies based on the host biomarkers are more accessible in light of the recent advances in the inflammation field. The effective prediction may prevent the development of IRIS, while clinical interventions using inflammatory modulation may improve outcomes in PLWH at risk for IRIS.

## Figures and Tables

**Table 1:** Summary of evidence for the use of host markers to predict IRIS.

Author	Year of Publication	Title of the Study	Type of IRIS/Site	Described Biomarkers	Ref
Breglio et al.	2020	Clinical and immunologic predictors of *Mycobacterium avium* complex immune reconstitution inflammatory syndrome in a contemporary cohort of patients with HIV	MAC-IRIS/Plasma	Increased expression of CD38 in CD8 ^+^ T cells High levels of alkaline phosphatase and D-dimer	[55]
Haddow et al.	2011	Circulating inflammatory biomarkers can predict and characterize tuberculosis-associated immune reconstitution inflammatory syndrome	TB-IRIS/Plasma	Reduced IL-10 and CCL2 levels Increased CRP and IFN-γ levels	[81]
Vinhaes et al.	2020	An inflammatory composite score predicts mycobacterial IRIS in people with HIV and severe lymphopenia: A prospective international cohort study.	TB-IRIS and Viral-IRIS/Plasma	A composite score including augmented levels of IL-6, IL-10, IL-27, sCD14, IFN-γ, TNF-α, Hyaluronic acid and D-dimer, and lower levels of hemoglobin	[90]
Andrade et al.	2013	Biomarkers of inflammation and coagulation are associated with mortality and hepatitis flares in persons coinfected with HIV and hepatitis viruses	HBV and HCV associated-IRIS/Plasma	D-dimer, IL-6, IL-8 and sCD14	[104]
Jarvis et al.	2015	Cerebrospinal fluid cytokine profiles predict risk of early mortality and immune reconstitution inflammatory syndrome in HIV-associated cryptococcal meningitis.	C-IRIS/Cerebrospinal Fluid	IL-6, IFN-g, IL-4, IL-10 and IL-27 Associated with protection	[121]
Boulware et al.	2014	Timing of antiretroviral therapy after diagnosis of cryptococcal meningitis	C-IRIS/Cerebrospinal Fluid	Decreased CSF leukocyte counts reduced levels of IFN-g, IL-6, IL-8 and TNF-a	[124]
Akilimali et al.	2017	Plasma, but Not Cerebrospinal Fluid Interleukin 7 and Interleukin 5 Levels Pre-Antiretroviral Therapy Commencement Predict Cryptococcosis-Associated Immune Reconstitution Inflammatory Syndrome.	C-IRIS/Plasma and Cerebrospinal Fluid	IL-7 and IL-5	[126]
Narendran et al.	2016	Role of LTA4H Polymorphism in Tuberculosis-Associated Immune Reconstitution Inflammatory Syndrome Occurrence and Clinical Severity in Patients Infected with HIV	TB-IRIS/DNA sample extracted from blood	LTA4H polymorphism	[141]
de Sá et al.	2020	Clinical and genetic markers associated with tuberculosis, HIV-1 infection, and TB/HIV-immune reconstitution inflammatory syndrome outcomes	TB-IRIS/DNA sample extracted from blood	Polymorphisms in HLA-B, HLA-C, and KIR2 DL3 genes	[142]
Price et al.	2002	Polymorphisms in cytokine genes define subpopulations of HIV-1 patients who experienced immune restoration diseases	Mycobacteria/Viral IRIS/DNA sample extracted from blood	TNFA-308*2 allele	[143]
Vlasova-St Louis et al.	2018	Transcriptomic Predictors of Paradoxical Cryptococcosis-Associated Immune Reconstitution Inflammatory Syndrome	C-IRIS/Plasma	Elevated expression of AIM2, BEX1 and C1QB	[144]
Silva et al.	2019	A pilot metabolomics study of tuberculosis immune reconstitution inflammatory syndrome	TB-IRIS/Plasma	Increased levels of epoxyeicosatrienoic acid, 15-deoxy-Δ-12,14-PGJ2, hydroperoxylinoleic acid and phosphatidylethanolamine. Reduced levels of phosphatidylcholine	[145]
Oliver et al.	2010	Mediators of innate and adaptive immune responses differentially affect immune restoration disease associated with Mycobacterium tuberculosis in HIV patients beginning antiretroviral therapy	TB-IRIS/Plasma	Diminished CCL2 levels	[146]
Tan et al.	2015	Plasma interleukin-18 levels are a biomarker of innate immune responses that predict and characterize tuberculosis-associated immune reconstitution inflammatory syndrome	TB-IRIS/Plasma	High IL-18 levels prior to ART	[147]
Musselwhite et al.	2016	Vitamin D, D-dimer, Interferon gamma, and sCD14 Levels are Independently Associated with Immune Reconstitution Inflammatory Syndrome: A Prospective, International Study	TB-IRIS and other-IRIS/Plasma	A composite score including CRP, sCD14, IFN-γ and hemoglobin	[148]
Mizukawa et al.	2020	Predictive biomarkers for cytomegalovirus reactivation before and after immunosuppressive therapy: A single-institution retrospective long-term analysis of patients with drug-induced hypersensitivity syndrome (DiHS)/drug reaction with eosinophilia and systemic syndrome (DRESS)	CMV- IRIS/Plasma	High baseline levels of IL-8, IL-10, IL-12p70, IL-15, G-CSF and CCL2	[149]

**Table 2:** Summary of evidence for the use of drugs related to prevent and treat IRIS.

Author	Year	Types of Study Designs	Type of IRIS	Country	Drug	Evidence	Ref
Meintjes et al.	2010	Randomized-controlled trial	TB-IRIS	Cape Town, South Africa	Prednisone	Prophylactic prednisone during the first 4 weeks after the initiation of ART in adult patients at high-risk for tuberculosis-associated IRIS resulted in a 30% lower incidence of tuberculosis-associated IRIS than placebo.	[151]
Stek et al.	2020	Randomized-controlled trial	TB-IRIS	Cape Town, South Africa		In severe TB-IRIS, the effect was ameliorated by treatment with prednisone, improved lung function at week 4, possibly by reducing TB-IRIS; however, the 28-day course of prednisone did not improve lung function from week 12.	[152]
Dooley et al.	2020	Randomized-controlled trial	TB-IRIS	There were 37 sites in 7 countries (Argentina, Brazil, Mexico, Peru, Russia, South Africa, and Thailand)	Dolutegravir	Tuberculosis-associated IRIS incidence was low in the Dolutegravir arm.	[155]
Sierra-Madero, et al.	2015	Randomized controlled trial	TB-IRIS	One site in South Africa and five in Mexico	Maraviroc	Maraviroc in an initial treatment regimen does not confer meaningful protection from the occurrence of IRIS.	[156]
Hachisu et al.	2019	Cohort study	TB-IRIS	Gunma, Japan	TNF-α inhibitor	The usage of TNF-α inhibitor was significantly associated with TB-IRIS development in non-HIV patients.	[157]
Keeley, et al.	2020	Case report	TB-IRIS	Patients from Ethiopian and Zimbabwe	Anakinra	Anakinra was used to achieve control of inflammation and to reduce and stop steroids in patients at risk of death or serious morbidity (in part due to high steroid requirements) with protracted paradoxical reactions to TB.	[158]
Jain, et al.	2016	Case report	TB-IRIS	India	Bevacizumab	The patient developed IRIS in the form of increased serous fluid, and they document its resolution with intravitreal bevacizumab.	[159]
Lwin, et al.	2018	Case report	TB-IRIS	Australia	Adalimumab	The case highlights the therapeutic effect of Adalimumab on IRIS without a negative impact on immunological and virological control of HIV infection in short-term follow-up.	[160]
Hsu, et al.	2016	Case report	TB-IRIS	Cameroon, Honduras and African American	Infliximab	In 3 patients with steroid-unresponsive mycobacterial IRIS, clinical improvement was temporally associated with the administration of infliximab without obvious adverse impact on immune recovery and HIV virologic control.	[161]
Brunel, et al.	2012	Case report	Cryptococcal meningitis-IRIS	France	Thalidomide	Two HIV-infected patients with CM showed rapid clinical remission and were able to stop corticosteroid treatment following treatment with thalidomide, allowing successful IRIS resolution	[162]
Lortholaryet al.	2005	Retrospective multicenter cohort	Cryptococcal meningitis-IRIS	France		Two patients received specific therapy for IRIS: thalidomide for 4 months, with a favorable progressive return to normal lymph-node size, with dramatic improvement within 10 days, and then lower doses for a total of 8 months.	[163]
. Rowley, et al.	2019	Case report	Hepatitis B virus–IRIS	Nigeria	HAART (emtricitabine, tenofovir and raltegravir)	Patient with hepatitis B virus-related IRIS, HAART was continued, and he was discharged on hospital day 12 after symptoms improved significantly.	[164]
Mitsumoto, et al.	2014	Case report	Hepatitis B virus–IRIS	Japan	ART: raltegravir, tenofovir disoproxil fumarate, emtricitabine	Two patients with hepatitis B virus/HIV IRIS continue ART. The alanine aminotransferase levels of both patients gradually decreased, and his HBV DNA and HIV RNA levels reduce.	[165]

## Abbreviations

PLWH	People living with HIV
ART	Antiretroviral therapy
OI	Opportunistic infections
Mtb	Mycobacterium tuberculosis
MAC	Mycobacterium avium complex
CMV	Cytomegalovirus
KSHV	Kaposi sarcoma associated herpesvirus
HCV	Hepatitis C virus
HBV	Hepatitis B virus
IRIS	Immune Reconstitution Inflammatory Syndrome
SI	Systemic inflammation
NICMs	Noninfectious comorbidities
IL	Interleukin
TNF	Tissue necrosis factor
CRP	C-reactive protein
TLR	Toll-like receptors
AIDS	Acquired Immunodeficiency Syndrome
HLA	Human leukocyte antigen
CXCR	chemokine receptor
LBP	Lipopolysaccharide-binding protein
CXCL	C-X-C motif chemokine ligand
CCL	C–C motif chemokine ligand
PD	Programmed cell death receptor
CTLA	Cytotoxic T-lymphocyte-associated protein
LAG	Lymphocyte-activation gene
OXPHOS	Oxidative phosphorylation
ATP	Adenosine triphosphate
GLUT	Glucose transporter
HAND	HIV-associated neurocognitive disorder
CKD	Chronic kidney diseases
CVD	Cardiovascular disease
TF	Tissue factor
PAR	Protease activated receptor
WHO	World Health Organization
MIP	Macrophage inflammatory protein
HIF	Hypoxia-inducible factor
IFN	Interferon
MMPs	Matrix metalloproteinases
TIMP	tissue inhibitors of metalloproteinases
TB-IRIS	tuberculosis-associated immune reconstitution inflammatory syndrome
M-IRIS	Mycobacterial IRIS
C-IRIS	Cryptococcus IRIS
VZV	Varicella-zoster virus
EOD	End-organ diseases
HHV	Human herpesvirus
CSF	Cerebrospinal fluid
PJP	Pneumocystis pneumonia
LTA4H	leukotriene A4 hydroxylase
KIR	killer-cell immunoglobulin-like receptors
BMI	Body mass index
DTG	Dolutegravir
INSPIRING	International Study of Patients with HIV on Rifampicin ING
